# The Global Polycrisis and Health Inequalities

**DOI:** 10.1177/27551938251317472

**Published:** 2025-02-16

**Authors:** Courtney McNamara, Clare Bambra

**Affiliations:** 1 Population Health Sciences Institute, Faculty of Medical Sciences, Newcastle University, Newcastle upon Tyne, UK

**Keywords:** social inequality, health disparities, socio-economic, economic, environment, syndemic, politics, global health, pandemics, crisis

## Abstract

In the current era of multiple, overlapping global crises, it is essential to consider the political economy of health within the broader framework of global interconnectedness. In this article, we employ the polycrisis concept to understand the impacts of the multifaceted, concurrent, and synergistic contemporary global crises on global health and health inequalities. A polycrisis occurs when crises in multiple diverse global systems become causally entangled, compounding their effects. Despite its potential relevance and analytical insights, the polycrisis concept has rarely been applied in public health research. This article fills that gap, and through reviewing the impacts of past economic, environmental, health, and political crises, we argue that the polycrisis is creating a complex web of challenges that are likely to amplify existing and future health inequalities. We conclude by discussing strategies to mitigate these impacts and suggest pathways for research to ensure that the future is not inevitably unequal.

The world has been navigating a polycrisis at least since the global financial crisis of 2008. In a polycrisis, global systems—such as those related to finance, climate, and health—are deeply interconnected and vulnerable to cascading disruptions, a seemingly isolated issue in one part of the world can quickly escalate into a far-reaching crisis. The global financial crisis of 2008, which began with defaults in the United States’ subprime housing market, soon spiraled into a worldwide economic downturn. Not long after, the 2010 eruption of the Eyjafjallajökull volcano in Iceland led to massive disruptions in air travel across Europe, affecting millions of passengers and global supply chains. Beginning in 2020, the COVID-19 pandemic not only caused widespread health and health care crises but also triggered a global economic downturn. More recently, Russia's invasion of Ukraine has severely disrupted global food supplies, particularly grains and fertilizers, leading to food security crises in many parts of the world.

Taking a political economy of health perspective, this article argues that the ongoing polycrisis has profound implications for global public health, shaping health inequalities now and for future generations. Reviewing evidence from the documented health inequalities impacts of economic (e.g., the global financial crisis of 2007–2008), environmental (e.g., Hurricanes Maria and Katrina), pandemic (e.g., pandemic flu, Zika, Ebola and COVID-19) and political (e.g., the rise of the populist radical right) crises, this article examines the likely health inequalities implications of the polycrisis. It examines how these multiple crises can interact to deepen existing inequalities and concludes by discussing potential strategies to mitigate the unequal impacts of this complex challenge for global health.

## The Political Economy of Health and the Global Polycrisis

There are several different approaches to health—biomedical, behavioral, social determinants, and political economy.^1^ The biomedical model focuses on possibilities for cures and views differences in health as differences among individuals; the behavioral approach focuses on what are widely referred to as lifestyle choices (smoking, alcohol consumption, healthy diets, seatbelt use) while normally neglecting the larger contextual influences (low incomes that make healthy diets unaffordable, intensive marketing of energy-dense convenience foods, etc.); and the social determinants of health focus on how the material environment influences people's health, such as housing, income, and employment. The political economy approach, by contrast, focuses on social, political and economic structures and relations that may be, and often are, outside the control of the individuals and communities they affect, asserting that health is politically determined.^2^

In the political economy approach, patterns of health and disease are “produced, literally and metaphorically, by the structures, values and priorities of political and economic systems. Health inequities are thus posited to arise from whatever is each society's form of social inequality, defined in relation to power, property and privilege”*,*^3^ p. 245). Another way of expressing this idea comes from work by Diderichsen and colleagues,^4^ who argued for explaining socially patterned inequalities in health in terms of how social stratification (the unequal distribution of resources and opportunities) generates differences in exposures to risks of illness, vulnerabilities to those exposures, and consequences of ill health. Further, they argued that explanations must venture upstream from the observed facts of stratification to consider “those central engines in society that generate and distribute power, wealth and risks” (p. 16). In 2005, Bambra and colleagues^2^ took a further step by putting forward the concept of the political determinants of health: “Health, like almost all other aspects of human life, is political in numerous ways . . . because, like any other resource or commodity under a neo-liberal economic system, some social groups have more of it than others . . . because its social determinants are amenable to political interventions . . . [and] because the right to ‘a standard of living adequate for health and wellbeing’ is, or should be, an aspect of citizenship and a human right. Ultimately, health is political because power is exercised over it as part of a wider economic, social and political system” (p. 2).

The political economy approach thereby argues that the behavioral, social and environmental determinants of health are themselves shaped by macro-level structural determinants and that health inequalities are thereby shaped by social, political, and economic structures, systems, institutions, and relations.^5^ Individual and collective social and economic factors such as housing, the environment, income, and employment—indeed many of the issues that dominate political life—are indirectly the key determinants of health and well-being.^2^ Health inequalities—between people and places—are thereby considered to be politically determined. Political action—or inaction—shaping key systems (e.g., economic policy, environmental policy, social policy) can thereby be seen as the “causes of the causes of the causes” of inequalities in health.^6^

The political economy approach to health has a long pedigree, arguably dating back to the nineteenth century (e.g., Rudolf Virchow 1821–1902;,^7^ with further influential work conducted in the 1970s (e.g.,^8^ and with seminal work throughout the 1980s, 1990s, and early 2000s—often published within this journal (e.g.,^9,10^).There has been a recent resurgence, particularly in relation to the examination of cross-national differences in health^11^ and within the analysis of the structural causes of inequalities in health, including racism (e.g.,^12^) economic policy (e.g.,^13^); social policies (e.g.,^14^) and power systems (e.g.,^15^).

In the current context of multiple, overlapping global crises, it is essential to consider the political economy of health within a broader framework of global interconnectedness. Developed by historian Adam Tooze^16^ and complexity theorists Edgar Morin and Anne Brigitte Kern,^17^ the concept of polycrisis captures this complexity. A polycrisis is said to occur when “crises in multiple global systems become causally entangled in ways that significantly degrade humanity's prospects. These interacting crises produce harms greater than the sum of those the crises would produce in isolation, were their host systems not so deeply interconnected”^18^ (p. 2). Lawerence and colleagues^19^ distinguish eight global systems that can become entangled in a polycrisis: the economy, health, social order and governance, food, international security, energy, environment, and transportation and communication. A crisis itself refers to a sudden event or series of events that causes significant harm to the well-being of a large population within a relatively short time frame. A crisis thus represents an emergency necessitating an urgent response to prevent greater harm.^19^ Further, crises can vary in scale from local to global, depending on their severity and the scope of their impact.^19^

The polycrisis concept does not simply denote the coexistence of multiple crises but highlights how these crises interact within complex systems and become synchronized. What may initially appear as discrete crises in fact alter and intensify one another, so that these causal interactions produce harms that are distinct from the harms they would have produced in isolation. Global warming, for example, is amplifying and accelerating zoonotic diseases, which have become increasingly severe and frequent. There are several ways crises across different global systems can interact, though the exact causal mechanisms of crisis interaction remain at the center of an emerging polycrisis research agenda.^19^

Constituent, ongoing crises of the current polycrisis include volatility in global food and energy markets, lingering health, social and economic effects of the COVID-19 pandemic, geopolitical conflicts, political instabilities, ideological extremism, and increasingly frequent climate events. These crises are undoubtedly diminishing humanity's prospects by their destruction of lives and livelihoods around the world.

Despite its relevance, the polycrisis concept has been used sparingly across health research. Authors Kalwak and colleagues^20^ have used it to study mental health among college students, finding that disadvantaged students exhibit greater vulnerability when faced with multiple concurrent crises. Their research suggests that when young adults show a psychological response to crises, it is often due to the simultaneous impact of multiple stressors rather than a single event. The authors suggest that this accumulation of stressors may mitigate other protective resources that would otherwise enable positive stress coping mechanisms. While this study demonstrates the value of a polycrisis perspective in understanding complex health challenges, most health-related work on the polycrisis has been limited to calls for action.^21–24^

While a polycrisis can occur at different levels (e.g., local, national, regional) here we consider how the convergence of global crises is likely to affect public health, contributing to a deeper understanding of health inequalities. The polycrisis concept relates to Singer's^25^ syndemic concept which has been used to examine the interaction of several health-related factors (e.g., COVID-19^26^), but adds essential novelty by allowing for an examination of how multiple crises across various global systems—for example, economic, health, environmental, and political—are interconnected and how they exacerbate and compound one another in a way that goes beyond individual or co-occurring health problems to include a fuller spectrum of global factors and their cascading effects.

Overall, there has been minimal prior application of the polycrisis concept in public health research. This article fills this void and is the first to utilize the polycrisis concept to evaluate global health and health inequalities. It begins by reviewing evidence of health inequalities in relation to different types of crises—economic, health, environmental, and political. It then applies the polycrisis concept within a political economy of health framework to argue that the current convergence of crises is creating a complex web of challenges that are likely to amplify existing and future health inequalities. We conclude by discussing potential strategies to mitigate these impacts and suggesting pathways for future research that can further explore the links between global crises and health inequalities.

## Economic Crises and Health Inequalities

Economic crises are a fundamental component of the polycrisis framework, as they frequently intersect with other types of crises and significantly impact public health. Since the global financial crisis of 2007–2008, major economies have experienced below average growth and economic volatility. This is especially the case since the COVID-19 pandemic and the Ukraine war with large increases in inflation and interest rates. These developments have increased the cost of living in many countries with sharp rises in the prices of food, energy and other necessities.

The global financial crisis of 2007–2008 was a result of a downturn in the U.S. housing market (largely driven by subprime investments), which led to a massive collapse in financial markets across the world. Banks increasingly required state bailouts (e.g., in the United Kingdom the retail bank Northern Rock was nationalized while in the United States, Lehmann Brothers investment bank filed for bankruptcy, and the mortgage companies Freddie Mac and Fannie Mae were given major government bailouts). Stock markets posted massive falls which continued as the effects in the “real” economy began to be felt with peak unemployment rates of over 8 percent in the United Kingdom and over 10 percent in the United States and the Euro-zone. In 2009, the International Monetary Fund (IMF) announced that the global economy was experiencing its worst period for 60 years.^27^ The global economic recession continued throughout 2009 and 2010 (leading to the moniker of global financial crisis).

Research has found that the global financial crisis lead to increases in suicides and mental ill health.^28^ For instance, a study found that the mental health of men in England deteriorated over the two years following the global financial crisis of 2007–2008.^29^ Mental health problems such as stress and depression were also found to increase during periods of recession in studies in Spain, Greece, and Ireland.^30–33^ There is also evidence of increases in self-harm and psychiatric morbidity.^34,35^ In a number of studies this was found to lead to an increase in mortality rates from suicide during periods of recession (e.g.,^36^) For example, following the 2007–2008 crisis, worldwide an excess of 4,884 suicides were observed in 2009 and over the next three years (2008–2010): an excess of 4,750 suicides occurred in the United States, 1,000 suicides in England, and 680 suicides in Spain.^37^

One of the main pathways whereby recessions adversely impact on health is through increased rates of unemployment. Unemployment is associated with worse mental health, including suicide.^38^ Areas with higher unemployment rates tend to have poorer neighborhood health outcomes, and at the country level increases in the unemployment rate have been associated with increased mortality.^39^ Some studies of previous economic downturns, including those in the 1970s, 1980s, and 1990s as well as the global financial crisis of 2007–2008, suggest that the unemployment—and therefore health—effects of economic downturns can be unequally distributed, thereby exacerbating health inequalities.^40^ For example, after the global financial crisis, areas of the United Kingdom with higher unemployment rates had greater increases in suicide rates, exacerbating health inequalities.^36^ Similarly, the gap in mental health and well-being between deprived and affluent areas in England increased as people living in more deprived areas bore the brunt of rising rates of mental ill health.^41^ The mortality rates of women in the most deprived areas of the United Kingdom also increased between 2010/2012 and 2017/2019,^42^ and life expectancy also declined in some of the most deprived areas. Similarly, a study of the impacts of the 2007–2008 financial crisis on inequalities in antidepressant use in Scotland found that people living in the local authority areas of Scotland most adversely economically impacted by the financial crisis had the highest risk of beginning a new course of antidepressants.^43^ People living in areas least impacted had the lowest risk. Emerging evidence from the post-COVID-19 economic downturn substantiate these findings.^44^

## Environmental Crises and Health Inequalities

The climate emergency is now widely accepted as the biggest public health crisis facing the planet.^45^ Exposure to drought and floods, heat and cold, hurricanes and typhoons, air pollution, pollen, food safety and security risks, disruptions to access to and functioning of health and utility services, global security, migration, and emerging infectious diseases are all key impacts of climate change, and all have the potential to adversely influence public health. The negative health impacts range from respiratory to mental health. Communities that are already disadvantaged are among the most vulnerable to the effects of these systemic environmental shocks and extreme weather events. Age, pre-existing medical conditions, gender, ethnicity and social deprivation are all factors that potentially make people more vulnerable to the adverse health impacts of climate change. Climate change therefore has the potential to widen existing health inequalities—within and between countries.

Floods are the most common type of disaster globally and often accompany hurricanes. The health effects of flooding include an increased risk of disease outbreaks (such as hepatitis E or gastrointestinal disease, particularly in low-income countries) and increased psychological distress.^46^ In terms of health inequalities, there is evidence from low-income countries that those at higher risk of flood-related death tend to be from ethnic minorities, poorer communities, and the very young and elderly. For example, a study of the 1993 flash flood in Nepal found that the mortality risk was higher among low socioeconomic status populations.^47^ In medium- and high-income countries, studies show that the elderly, poorer communities, and minority ethnic groups experience more flood-related casualties compared with other communities.^48,49^ For example, analysis of flood-related casualties in eastern Texas revealed that the risk for death or injury was higher in communities with more socially vulnerable populations.^50^

Heatwaves (such as those experienced in Europe in 2003 and globally in 2022) lead to increased mortality and morbidity rates.^51^ For example, an Australian study of heatwaves between 1988 and 2011 in Adelaide, Brisbane, Melbourne, Perth, and Sydney found that the mortality rate increased by 28 percent in the short term.^52^ Similarly, Korean and Iranian studies have found that the overall mortality risk increased by over 11 percent.^53,54^ Other studies, such as those conducted in Finland and China, have found significant effects of heatwaves on cardiovascular mortality.^55–57^ International research has also found significant inequalities in these health effects with older people and people from lower socioeconomic groups or with prior health conditions (e.g., cardiopulmonary diseases, renal disease, diabetes) being particularly negatively affected.^51^ Low socioeconomic status was also significantly associated with heatwave-related morbidity and increased emergency department visits in Australia.^57^

Hurricanes are also associated with adverse health impacts and health inequalities. Research into Hurricane Katrina (a devastating category 5 Atlantic hurricane in late August 2005, particularly affecting the city of New Orleans and its surrounding areas) and Hurricane Maria (a deadly category 5 hurricane that devastated the northeastern Caribbean in September 2017, particularly Dominica, Saint Croix, and Puerto Rico) has found substantial inequalities in their health impacts. It is estimated that between 1,300 and 1,800 fatalities resulted from Hurricane Katrina. Black Americans were overrepresented among fatalities above the age of 18, with a mortality rate up to four times higher than that of white Americans.^58^ Research has also found that there were substantial inequalities in cardiovascular disease hospitalizations during the hurricane and the subsequent floods; a week after the hurricane, hospitalization rates increased to 26.3 and 16.6 cases/day per 10,000 people for black and white patients, respectively.^59^ Other key findings in terms of health inequalities include: black hurricane survivors more frequently reported hurricane-related problems with health, emotional well-being, and finances^60^; displaced persons were more likely to be female, black, low-income, without health insurance and suffering from chronic disease^61^; and people who experienced socioeconomic decline (such as unemployment or poverty) post-hurricane were more likely to experience adverse health outcomes (including elevated risk of a cardiometabolic event and chronic pain).^62^ Likewise, research by George Washington University^63^ has estimated that excess mortality in Puerto Rico attributable to Hurricane Maria was 2,975. While every social stratum and age group was affected by excess mortality, the impact differed; risk of death was higher and more persistent for populations living in low socioeconomic development municipalities (around 45% higher than the most developed).

## Pandemic Crises and Health Inequalities

Pandemic crises have clear implications for public health and health inequalities. The frequency and scale of emerging infectious diseases (EIDs) with pandemic potential has been increasing over the last two decades and, as COVID-19 has shown, such zoonotic spillover events are an increasing threat to public health globally. Since 2007, the World Health Organization (WHO) has made six Public Health Emergency of International Concern (PHIEC) declarations: the 2009 H1N1 influenza pandemic, Ebola (West Africa 2013–2015, Democratic Republic of Congo 2018–2020), poliomyelitis (2014 to present), Zika (2016) and COVID-19 (2020 to present).^64^ In this section, we provide an overview of inequalities in the health impacts of previous pandemics (focusing on Spanish flu, H1N1, Zika, Ebola, and COVID-19.^65^

In 1918, the world experienced a major global pandemic: Spanish flu. Recent historical research has demonstrated that there were clear socioeconomic and geographical inequalities in the impact of the Spanish flu pandemic.^66^ Infection and death rates were substantially higher in less affluent neighborhoods, amongst the working classes and lower paid workers, and in urban areas. In Norway death rates were highest in the working-class districts of Oslo^67^; in the United States they were highest amongst the unemployed and the urban poor^68^; in Australia death rates were lower among professional and commercial groups and higher in lower status occupations, such as laborer^69^; in Spain they were highest amongst low income groups^70^; in England and Wales they were higher in more industrialized areas^71^ and across London, there was a clear association between influenza mortality and household wealth^72^; in Sweden and The Netherlands, deaths were higher in the lowest occupational classes.^73,74^ Research into ethnic inequalities in the 1918 pandemic in the United States has found that black Americans had lower morbidity and lower mortality than white Americans, but a higher case fatality rate.^75^

In the spring of 2009, a novel influenza A (H1N1) virus emerged. Inequalities were also evident in this pandemic. The mortality rate in the most deprived 20 percent of England's neighborhoods was three times higher than in the least deprived 20 percent.^76^ Similarly, in Canada, hospitalization rates for H1N1 were associated with lower educational attainment and living in a high deprivation neighborhood.^77^ In the United States, people with financial problems (e.g., financial barriers to health care access) were more likely to report H1N1 symptoms.^78^ Research conducted in Europe and the Americas found evidence that minority ethnic and indigenous groups experienced an increased mortality risk.^79–83^

The Ebola (2015–2016, 2018–2020), and Zika (2016) pandemics also resulted in socioeconomic inequalities in terms of morbidity and mortality.^65^ Ebola research in West Africa has found that transmission was 50 percent higher in the most impoverished communities and that most of the spread originated in lower socioeconomic status areas.^83^ Likewise, research into the Zika pandemic in Brazil and the Americas has found strong associations between the resulting microcephaly and living conditions; populations with the worst living conditions had a prevalence ratio for microcephaly more than five times higher than those living in areas with the best living conditions.^84^

In December 2019 the first cases of an unusual pneumonia were documented in the Chinese city of Wuhan. The novel disease, which seems to have jumped from an animal population into humans, was later named SARS-CoV-2, or COVID-19 (coronavirus disease 2019). By late January 2020, the WHO declared COVID-19 a “public health emergency of international concern.” COVID-19 is now a global phenomenon, affecting all parts of the world and all parts of society, and during the first two years, it radically altered how we live and interact. Everyone, from all walks of life, was affected by the pandemic. However, some people have been—and will be—far more affected than others, as COVID-19 is an unequal pandemic.^85^ There is now substantial evidence that deaths from COVID-19 are up to three times higher in more deprived neighborhoods, amongst people with low incomes, in urban compared to rural areas, and among some minority ethnic groups.^85^ Emergency measures taken to contain the virus, including lockdowns, also impacted people unequally.^85^

## Political Crises and Health Inequalities

Current political instabilities in the world also have implications for health inequalities. Ongoing wars in Gaza, Ukraine, Sudan, Myanmar, and elsewhere will directly impact health inequalities through conflict and state fragility. It is widely recognized, for example, that women suffer differential effects in both conflict and post-conflict settings, including through sexual violence.^86^ Other groups that are especially vulnerable in war settings include children and those who are internally displaced.^87^ In the ongoing conflict in Syria, for example, children have been found to be twice as likely to die from chemical weapon attacks as adults.^88^ There is also evidence suggesting that the mortality rate of children under five years old nearly doubles among forcibly displaced children compared to their nonmigrating counterparts.^89^

State conflicts not only exacerbate health inequalities but also signal a world that is becoming increasingly unstable, divided, and drifting toward illiberal forms of politics. The ascent of the populist right, marked by its anti-immigration, culturally conservative, and nationalistic ideologies, is a significant outcome of this global shift, with numerous consequences for health and health inequalities.^90^

Populist radical right parties—including the *Rassemblement National* (French National Front), the Austrian Freedom Party (FPÖ), the Italian Northern League, the Alternative for Germany (AfD), the Polish Law and Justice (PiS) party, the Dutch Party for Freedom (PVV), the True Finns party, and the Sweden Democrats—are increasingly gaining political power. They are nationalist/nativist, authoritarian, and populist (privileging the “common sense” of “the people” over elite knowledge.^91^

Populist radical right ideology has most visibly influenced mainstream right-wing parties including the United Kingdom's Conservative Party since the 2016 Brexit vote and Republicans in the United States under Donald Trump. A central component of this ideology among the Conservative party in the United Kingdom is “welfare chauvinism”—increasing or defending welfare provisions (notably social security and health care) for the native-insider population while limiting access and eligibility for outsider groups, most notably immigrants and ethnic, religious, cultural, and linguistic minorities.^92^ Welfare chauvinism links native birth or ethnicity (and sometimes other attributes related to religion, culture, and language) to moral “deservingness,” which entitles those who possess it—and only those—to state support in time of need.^93^

There are clear—and morally challenging—implications of the linkage of nativity with deservingness for the health of minority groups.^93^ Minority ethnic groups often have worse health than the native population; for example, in many countries, they have higher rates of hypertension, diabetes, asthma, heart, liver, and renal disease, cancer, cardiovascular disease, obesity, and smoking.^94^ And yet, the influence of populist radical right welfare chauvinism has led to calls for—and in some countries such as the United Kingdom implementation of—restrictions on access to health care and welfare state support for immigrant communities.^93^ This has huge public health implications, not only for the health of the excluded population groups but also, in the context of endemic infectious diseases (notably tuberculosis and COVID-19), for the entire population.

In the case of Republicans in the United States, there has been a broader push to restrict welfare access for all groups, even those traditionally included within the native-insider category (e.g., white citizens). This goes beyond welfare chauvinism and reflects a more generalized retrenchment of welfare provisions.

Other notable areas of public health policy that have been beneficial for reducing health inequalities are currently under threat from populist radical right parties, including tobacco control (e.g., the Austrian coalition government incorporating the Austrian Freedom Party canceled the planned public smoking ban) and reproductive health rights (e.g., in the United States, the restriction of access to abortions and birth control).^95^ More generally, the wider populist radical right agenda rejects identity politics and the rights of LGBTQ + minorities^92^ and is skeptical of climate change. These ideological and policy stances forecast a concerning trajectory for health inequalities in terms of perpetuating societal inequalities and impeding progressive policies aimed at addressing them.

## Applying the Polycrisis Concept Within a Political Economy of Health Framework

A polycrisis framework emphasizes that crises across different global systems, though seemingly distinct, are deeply interconnected and often occur simultaneously or consecutively, leading to overlapping and compounding effects. Current geopolitical tensions, for instance, are fueling economic pressures, keeping energy prices high, perpetuating high costs of living, and contributing to global food insecurity.^96^ War and conflict often precede economic collapse, fostering social instability and triggering migration surges. Displacement, in turn, can severely impact ecosystems, and the long-term effects of conflict on economics and climate change may increase country fragility and the risk of conflict recurrence. Health and climate crises also go hand in hand. Evidence suggests that changing weather patterns can drive species to higher altitudes, exposing them to new diseases that increase the potential for human transmission.^97^

A polycrisis perspective indicates that these crises are not only interconnected but can exacerbate one another. Countries’ vulnerability to climate change, for instance, intensifies amid economic stress, while environmental disasters and economic downturns compound poverty and amplify health risks for vulnerable communities.^98^

Applying the polycrisis concept within a political economy of health framework suggests that people, especially vulnerable populations, are likely experiencing multiple crises at once or back-to-back, with each crisis amplifying the effects of the others. Existing research on health inequalities, however, often focuses on the impacts of isolated crises. There is thus an urgent need for more precise research that considers the compounded nature of crises to understand their full impact on health inequalities.

The polycrisis perspective also underscores the need to look at health inequalities from a multidimensional perspective that includes political, environmental, and economic contexts, recognizing that inequalities are not static but evolve as crises converge and interact. This approach offers a more dynamic understanding of health inequalities as a product of intersecting global systems.

Polycrisis analyst Zack Walsh points out that it is crucial to understand the drivers of the polycrisis, particularly the unjust power relations that shape these crises.^99^ This idea aligns directly with the political economy of health framework, which emphasizes that health inequalities are fundamentally shaped by broader political and economic structures. Crucially, while health equity is often seen as an outcome of these broader social, political, and economic processes, there is growing evidence that addressing health equity itself can be a powerful driver for transforming these systems.^100^

However, crisis dynamics have worsened markedly over the last three decades, and it is expected that this negative trend will persist. Ongoing conflicts and geopolitical fragmentation, for example, could lead to higher price volatilities with potentially large macroeconomic impacts.^101^ Further, the most recent U.N. climate report indicates that this last decade was the warmest on record and that since 1990 each successive decade has been increasingly warmer. More sobering, the Intergovernmental Panel on Climate Change has warned that the time to secure a livable and sustainable future for all is rapidly diminishing.^102^

## Does the Global Polycrisis Mean that Our Future Health is Inevitably Unequal?

In this context, the polycrisis concept prompts reflection on the inevitability of future health inequalities. By examining how multiple crises intersect and amplify each other, the polycrisis concept draws attention to the underlying systemic vulnerabilities that make societies more susceptible to cascading impacts. These systemic vulnerabilities are evident in the persistence of health inequalities, which are fundamentally rooted in broader social inequalities. Although numerous global examples illustrate how governments have addressed social inequality through welfare state expansion, improved health care access, and enhanced political incorporation, the polycrisis perspective reflects a profound confluence of global interdependencies, differentiating it from past challenges.^103^ In a polycrisis, decisions made in one corner of the world reverberate across continents, affecting distant communities and creating complex feedback loops that exacerbate existing inequalities.

Addressing the unequal health impacts of the polycrisis thus requires critical scrutiny of global interconnections and the policies, actors, and processes driving them. One of the main drivers of global connections is governments’ unwavering pursuit of economic growth. Addressing the weaknesses of this nearly singularly focused pursuit is crucial for substantial progress to be made in reducing health inequalities in times of polycrisis. In part, this will entail redefining government success beyond economic metrics and emphasizing comprehensive societal well-being as the primary objective of government policy.

While this shift in priorities may seem ambitious, the idea that government policies should prioritize not growth alone but also broader well-being goals like public health, social equity, and sustainable development^104–107^ is gaining traction. The WHO, for example, is an active champion of “well-being economies” and globally, individual countries like New Zealand and Finland have developed plans to measure and prioritize societal well-being within their economies, offering a potential inclusive approach to economic and social progress.^108,109^

It has been argued that the polycrisis is not a crisis of capitalism, as capitalists are doing rather well, but a crisis of what was promised by it. As Michael Jacobs^110^ has aptly pointed out: “The promise of the free-market revolution initiated forty years ago by Thatcher and Reagan was that it would make people better off: deregulating financial and labour markets; cutting income, corporation and capital taxes; restricting the power of trade unions to enable a ‘flexible’ labour market; reducing the size and role of the state in public services. All this would free up private enterprise to generate growth and jobs, and the extra profit and wealth would trickle down to everyone. But it hasn’t” (p. 4).

This realization has led like-minded political and economic scholars, as well as institutions outside of the health sphere, to advocate for a shift in government priority-setting beyond mere economic growth, demonstrating more widespread support for this approach.^111,112^ While there is still criticism regarding this idea (see, e.g.,^113^), examining health inequalities through a polycrisis perspective provides additional justification for this approach. A reorientation of priorities, where societal and environmental goals take precedence over economic objectives, offers a pathway where health inequalities need not be inevitable.

Engaging in robust debate on these ideas is imperative, but it is paramount that collective efforts drive immediate and tangible change toward this end. By embracing a polycrisis framework, health researchers can help inform policies that address the cumulative effects of interacting crises and support the inclusion of health equity as a central goal in policymaking.

## Conclusion

Across different types of rolling, cumulative, global crises and whole system catastrophic shocks, health inequalities are exposed and amplified with evidence that people in lower socioeconomic groups, people with disabilities, and people from minority ethnic backgrounds are more adversely affected by these events. While there is evidence of how governments can successfully reduce health inequalities, a feature that distinguishes the polycrisis from these earlier challenges is its roots in global interconnectedness. The polycrisis concept requires us to critically consider the drivers of global interlinkages and the economic priorities driving them. A reorientation of political priorities, whereby societal and environmental goals take precedence over economic objectives is vital if we are to avoid health inequalities as an inevitable fallout of the polycrisis.
